# Estimation of hereditary fructose intolerance prevalence in the Chinese population

**DOI:** 10.1186/s13023-022-02487-3

**Published:** 2022-08-26

**Authors:** Meiling Tang, Xiang Chen, Qi Ni, Yulan Lu, Bingbing Wu, Huijun Wang, Zhaoqing Yin, Wenhao Zhou, Xinran Dong

**Affiliations:** 1 Center for Molecular Medicine, Children’s Hospital of Fudan University, Shanghai, China; 2 Department of Neonatology, Children’s Hospital of Fudan University, Shanghai, 201102 China; 3 Department of Pediatrics, Dehong Hospital of Kunming Medical University, Dehong, 678400 China

**Keywords:** Hereditary fructose intolerance, Prevalence estimation, Curation for pathogenic variants, Newborn screening, Allele frequency comparison

## Abstract

**Background:**

Hereditary fructose intolerance (HFI) caused by aldolase B reduction or deficiency that results in fructose metabolism disorder. The disease prevalence in the Chinese population is unknown, which impedes the formulation of HFI screening and diagnosis strategies.

**Materials and methods:**

By searching a local cohort (Chinese Children’s Rare Disease Genetic Testing Clinical Collaboration System, CCGT) and public databases (ClinVar and Human Gene Mutation Database) and reviewing HFI-related literature, we manually curated *ALDOB* pathogenic or likely pathogenic (P/LP) variants according to ACMG guidelines. Allele frequency (AF) information from the local database CCGT and the public databases HuaBiao and gnomAD for *ALDOB* P/LP variants was used to estimate and the HFI prevalence in the Chinese population and other populations by the Bayesian framework. We collected the genotype and clinical characteristics of HFI patients from the CCGT database and published literature to study genotype–phenotype relationships.

**Result:**

In total, 81 variants of *ALDOB* were curated as P/LP. The estimated Chinese HFI prevalence was approximately 1/504,678, which was much lower than that for non-Finland European (1/23,147), Finnish in Finland (1/55,539), admixed American (1/132,801) and Ashkenazi Jewish (1/263,150) populations. By analyzing the genetic characteristics of *ALDOB* in the Chinese population, two variants (A338V, A338G) had significantly higher AFs in the Chinese population than in the non-Finland European population from gnomAD (all *P* values < 0.05). Five variants (A150P, A175D, N335K, R60*, R304Q) had significantly lower AFs (all *P* values < 0.1). The genotype–phenotype association analyses were based on 68 reported HFI patients from a literature review and the CCGT database. The results showed that patients carrying homozygous variant sites (especially A150P) were more likely to present nausea, and patients carrying two missense variant sites were more likely to present aversion to sweets and fruit (all *P* values < 0.05). Our research reveals that some gastrointestinal symptoms seem to be associated with certain genotypes.

**Conclusion:**

The prevalence of HFI in the Chinese population is extremely low, and there is no need to add HFI testing to the current newborn screening programs if medical costs are considered. A genetic testing strategy is suggested for early diagnosis of HFI.

**Supplementary Information:**

The online version contains supplementary material available at 10.1186/s13023-022-02487-3.

## Introduction

Hereditary fructose intolerance (HFI) is a rare inherited autosomal recessive (AR) disease caused by pathogenic variants of the aldolase enzyme, B isoform gene (*ALDOB*), which leads to the fructose-1,6-bisphosphate aldolase B (aldolase B) reduction or deficiency that results in fructose metabolism disorder [1]. Fructose is a monosaccharide similar to glucose that is the dominant component of candy, fruits and honey. It is also a metabolic intermediate of sucrose and sorbitol. Therefore, patients with HFI develop symptoms when they are exposed to fructose, sucrose or sorbitol [2]. The predominance of the liver, kidney, and small intestine in fructose metabolism is based on the presence of three enzymes (fructokinase, aldolase B and triokinase) that convert fructose into intermediates of the glycolytic–gluconeogenic pathway (fructose pathway) [3]. It is first phosphorylated to fructose-1-phosphate (F-1-P) by fructokinase, then converted into triose-phosphate by aldolase B [3–5]. Deficiency of aldolase B leads to the toxic accumulation of F-1-P and causes multiple clinical manifestations, including nausea, vomiting, hypoglycemia, metabolic acidosis, liver dysfunction, and abnormal renal function [6]. As a special diet (withdrawal of all sources of fructose, such as food, drugs, and parenteral infusions) helps prevent severe complications, early diagnosis can greatly improve the prognoses of patients, and serious complications can be avoided.

The prevalence of HFI is approximately 1:23,000 in Britain, and 1:20,000 in Switzerland [7], 1:26,100 in Germany [8], 1:31,000 in Poland [9], 1:34,483 in Northwest Russia and so on [10]. In general, the prevalence of HFI in European populations is approximately 1:31,000 to 1:18,000 [11]. The HFI prevalence in the Chinese population remains unknown [11, 12].

To date, the relationship between genotype and phenotype in HFI patients is unknown. Davit-Spraul et al. studied 162 patients from 92 families with HFI and found 16 mutations in the *ALDOB* gene. They collected the clinical symptoms of 10 probands, performed conventional tests (fructose load, hepatic aldolase B activity detection), identified variant sites, and recorded patients’ age, family history and so on. There was no significant genotype/phenotype correlation in the 10 families [13]. Mehmet Gunduz et al. retrospectively analyzed a cohort of 26 HFI patients [6]. They recorded the patients’ age, clinical symptoms, metabolic crisis history and variant sites. They also found no clear correlation between genotype and phenotype. Previous studies generally reported that no genotype–phenotype correlations were evident for HFI and that clinical severity and the extent of organ damage appeared to depend on an individual's nutritional environment [14]

After a literature review, we found that different populations had different pathogenic hotspots in *ALDOB*. The missense mutations A150P and A175D are the two most common alleles in the United States of America, Germany, Italy, the United Kingdom, France, Poland, etc., whereas A175D and R60* in Poland, A150P and N120Kfs*32 in Spain, and N335K and A150P in Australia are the most common alleles in those countries. The situation for the Indian population is very complex, and several variants with high allele frequency (AF), including c.112 + 1delG, c.324 + 1G > A, and c.380–1 G > A, have been identified [15]. The AF characteristics of pathogenic or likely pathogenic (P/LP) *ALDOB* variants in the Chinese population are unclear. Thus, we aimed to estimate the prevalence of HFI in the Chinese population, summarize the genotype–phenotype correlation, analyze the genetic characteristics of *ALDOB* and evaluate the necessity of newborn screening in the Chinese population. Therefore, in this study, we estimated the prevalence of HFI and analyzed the hotspots of *ALDOB* in Chinese population and to evaluate whether HFI screening in newborn babies is necessary. We also tried to expand the sample size and simultaneously study the relationship between variant site, mutation type, zygosity and phenotype in HFI patients. Our research is expected to be conducive to genetic counseling and to provide useful information for neonatal genetic screening.

## Results

### Curation of pathogenic variants of the ALDOB gene and allele frequency analysis

**Fig. 1:**
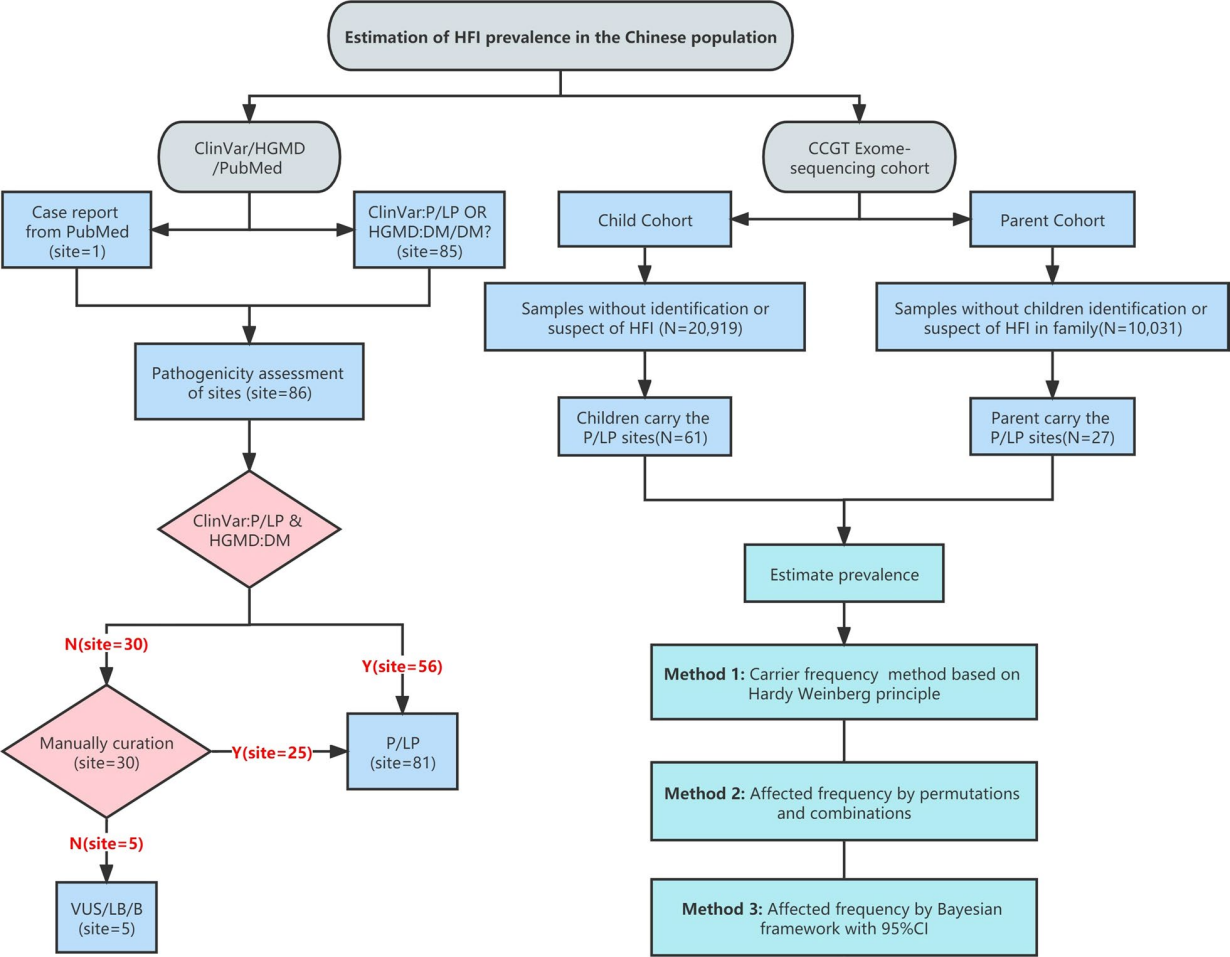
The workflow for estimation of HFI prevalence in the Chinese population. This study consisted of two parts were performed: (1) *ALDOB* variant pathogenicity curation. (2) Estimation of HFI prevalence

**Fig. 2:**
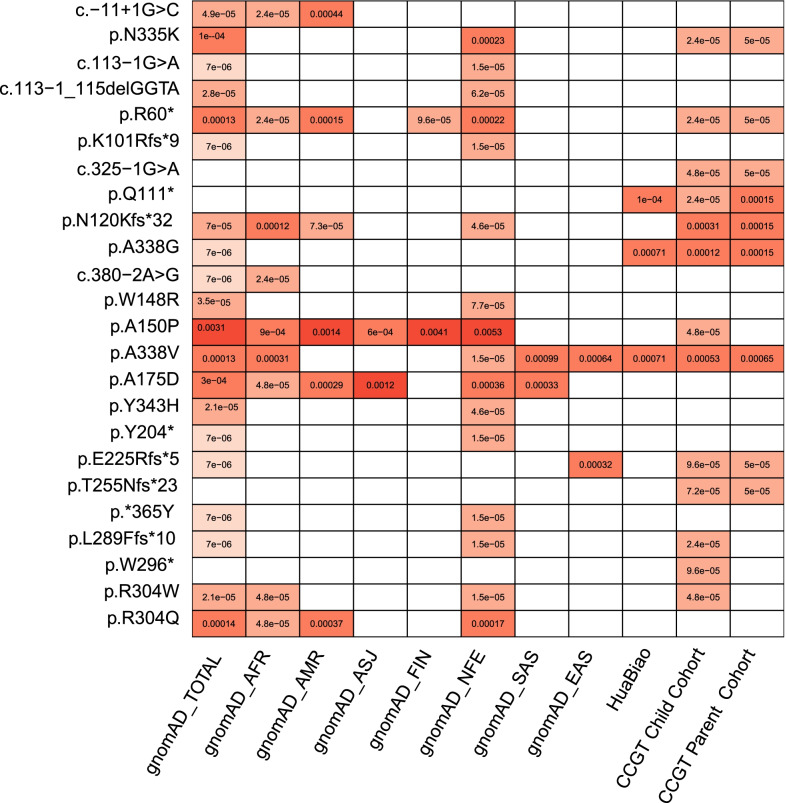
Allele frequency comparison for 24 pathogenic variants to different populations. The value in each box shows the AF for each variant (row) in each population (column). The blank box means the variant has not been detected in the population. All variants will be show if there are less than 10 variants in the mutation type. In total 13 P/LP variants had AF higher than 1e-5 in local CCGT database, and the A338V were significantly higher compared with the other populations (all *P* values < 0.05). AFR: African American; ASJ: Ashkenazi Jewish; NFE: non-Finland European population; FIN: Finnish in Finland; AMR: admixed American population; SAS: South Asian; EAS: East Asian population

After pathogenicity assessment of *ALDOB* variants identified in public databases, including ClinVar, Human Gene Mutation Database (HGMD) and PubMed (no additional findings in Web of Science), 81 P/LP variants were identified (Fig. 1, Additional file 1: Table S1), including missense (26%, 21/81), frameshift (22%, 18/81), splicing (21%, 17/81), nonsense (15%, 12/81), in-frame indel (6%, 5/81), indel (2%, 2/81), CNV (2%, 2/81), stop lost (1%, 1/81), start lost (1%, 1/81), synonymous (1%, 1/81) and 3’-UTR (1%, 1/81) variants. Among the 81 P/LP variants, 24 had reported AF information for general populations in public databases, including HuaBiao and gnomAD (Fig. 2, Additional file 1: Table S2). The HuaBiao database is the first whole-exome public database of heathy individuals of the Han Chinese population in China [16]. GnomAD collects exome and genome sequencing data of healthy individuals from various races. Therefore, we analyzed the AF information for these variants in different populations to detect the genetic characteristics of *ALDOB* in the Chinese population. In total, two variants (A338V, A338G) had significantly higher AF values in the Chinese population than in the non-Finland European population (all *P* values < 0.05), and five P/LP variants (A150P, A175D, N335K, R60*, R304Q) had significantly lower AF values (all *P* values < 0.01) in the Chinese population. The most frequent P/LP site in the gnomAD-TOTAL (all populations in gnomAD) is A150P (1:323), and it is also the most frequent site in admixed American (1:714), Finnish in Finland (1:244) and non-Finland European (1:189) populations. The hotspot detected in this study is consistent with a previous study [15]. However, the frequency is low in the Chinese Children's Rare Disease Genetic Testing Clinical Collaboration System (CCGT) Child Cohort (1:20,833). CCGT is an internal database and one of the largest exome genetic databases of the Chinese population. CCGT consists of patient information for children suspected of having genetic diseases and the information for their healthy parents. In addition, A150P was not recorded in the CCGT Parent Cohort or the HuaBiao database. A338V is the most common variant site in the Chinese population (HuaBiao, 1:1408; CCGT Child, 1:1887; and CCGT Parent, 1:1538), and it is also the most frequent site in African American (1:3226), South Asian (1:1010) and East Asian (1:1563) populations. This variant has not been reported in Ashkenazi Jewish, Finnish in Finland and admixed American populations. A338G is the second most frequent pathogenic site in the Chinese population (HuaBiao, 1:1408; CCGT Child, 1:8333; and CCGT Parent, 1:6667). N120Kfs*32 is the third most frequent variant in the Chinese population (CCGT Child, 1:3226 and CCGT Parent, 1:6667). The AF of N120Kfs*32 in the Chinese population is slightly higher than that in African American (1:8333), admixed American (1:13,699) and non-Finland European (1:21,739) populations (all *P* values > 0.05). These results showed that the *ALDOB* variants of the Chinese population have special characteristics different from those of a Caucasians (non-Finland European) population. Therefore, if it is necessary to quickly screen pediatric patients in China, the ten high-frequency sites we recommend are A338V, N120Kfs*32, A338G, W296*, E225Rfs*5, R304W, A150P, c.325-1G > C, L289Ffs*10 and Q111*. These variants were identified as the top 10 variants with high AF in the CCGT Child Cohort and covered approximately 90% of Chinese pediatric patients (Additional file 2: Fig. S1).

### Estimation of HFI prevalence in the Chinese population

We estimated the HFI prevalence based on 20,919 pediatric patients in the CCGT Child Cohort (12,783 males and 8136 females) and 10,031 parental samples in the CCGT Parent Cohort (5006 males and 5024 females). The total number of individuals carrying P/LP *ALDOB* variants was 61 (36 males and 25 females) in the Child Cohort and 27 (11 males and 16 females) in the Parent Cohort. Based on these results, the estimated HFI prevalence in the Chinese population was between 1/470,416 and 1/462,233 in the Child Cohort and 1/571,594 and 1/532,412 in the Parent Cohort. Specifically, the estimated prevalence of HFI by the Bayesian framework was 1/462,839 (95% confidence interval 1/803,692 ~ 1/293,567) in the Child Cohort and 1/532,412 (95% confidence interval 1/1,270,634 ~ 1/277,464) in the Parent Cohort. In total, the estimated prevalence of HFI in the Chinese population is 1/504,678 by averaging all the above results (Table 1). The prevalence rates in the HuaBiao database (1/410,068) and the East Asian population (1/818,758) are similar (Additional file 2: Table S7).

**Table 1:** HFI prevalence estimation in Child Cohort and Parent Cohort with estimated affected frequency by three methods

	CCGT child cohort	CCGT parent cohort
Total number	20,919	10,031
Gender (male/female)	12,783/8136	5006/5024
Carrier with P/LP variants (male/female)	61 (36/25)	27(11/16)
Carrier frequency	1/342	1/371
Couple’s carrier risk	1/117604	1/138026
Method 1: carrier frequency	1/470416	1/552104
Method2: permutation and combination	1/462233	1/571594
Method 3: Bayesian framework (95% CI)	1/462839 (1/803692 ~ 1/293567)	1/532412 (1/1270634 ~ 1/277464)
Average	1/465133	1/551573
Estimated HFI frequency	1/504678	

**Fig. 3:**
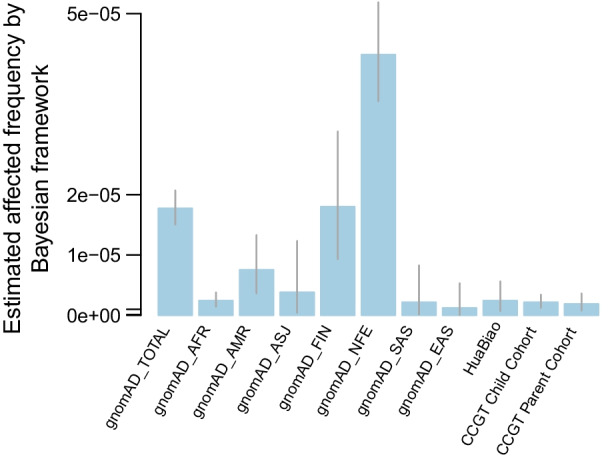
Estimated HFI affected frequency to different populations by Bayesian framework. Estimated affected frequency on 81 P/LP screening panel by Bayesian framework. Each population has a bar with each showing the estimated affected frequency if only use the pathogenic variants in the panel, and the *P* value above shows the significance for difference. The vertical line shows the 95% confidence interval. AFR: African American; ASJ: Ashkenazi Jewish; NFE: non-Finland European population; FIN: Finnish in Finland; AMR: admixed American population; SAS: South Asian; EAS: East Asian population

For the HFI prevalence estimation by the Bayesian framework in other gnomAD populations, the prevalence was 1/23,147 (95% confidence interval 1/28,182 to 1/19,278) in non-Finland European, 1/55,539 (95% confidence interval 1/107,444 to 1/32,816) in Finnish in Finland, 1/132,801 (95% confidence interval 1/274,861 to 1/75,230) in admixed American, 1/263,150 (95% confidence interval 1/2,275,631 to 1/81,267) in Ashkenazi Jewish, 1/412,335 (95% confidence interval 1/702,621 to 1/264,931) in African American and 1/465,278 (95% confidence interval 1/7,821,624 to 1/121,173) in South Asian populations (Fig. 3, Additional file 2: Table S7). Based on these results, we found that the prevalence in the Chinese population was much lower than that in other populations, especially in a Caucasian (non-Finland European) population.

### Diagnosed children in the CCGT database

We diagnosed three HFI patients in the CCGT database. Patient 1 was feeding with infant formulas after birth, approximately half an hour she presented vomiting. Hunger and poor complexion occurred after eating sweets. Since her parents stopped feeding her fructose-related foods at three years old, she did not present fructose-related gastrointestinal phenotypes. She could speak at seven months old and walk at two years old. She is now 174.6 cm, and her BMI is 18.4. The diagnosis was confirmed by genetic testing at the age of 30. She had compound heterozygous variants in the *ALDOB* gene: one pathogenic missense (c.1013C > T, p.A338V) variant from the father and one frameshift (c.360_363delCAAA, p.N120Kfs*32) variant from the mother [17]. Patient 2 was a one-year-old girl. She was breastfeeding soon after birth. At 8 months old, she began mixed feeding of an infant diet and breastfeeding. Vomiting occurred within half an hour after eating the infant diet. Her mother stopped feeding her the infant diet, and the vomiting was relieved. The genetic diagnosis was confirmed by genetic testing at the age of 8 months. Gene analysis revealed a pathogenic nonsense (c.888G > A, p.W296*) variant from the father and a missense (c.1013C > T, p.A338V) variant from the mother. After early dietary guidance, her abnormal liver function tests and hepatomegaly disappeared when she was 10 months old. Patient 3 was a three-year-old girl. After a week of breastfeeding, she presented jaundice, lethargy, metabolic acidosis, anemia, and thrombocytopenia and was diagnosed with acute liver failure. Genetic testing showed compound heterozygous variants in *ALDOB*, namely, a pathogenic frameshift (c.673_674delinsA, p.E225Rfs*5) variant and a splice acceptor (c.325-1G > A) variant. She remained healthy with normal growth and development at the time of the interview (3 years old). Based on clinical symptoms and genetic test results, all three patients were diagnosed with HFI. They did not present poor prognoses and developed well after diet control.

### The genotype–phenotype relationship in 68 HFI patients

In total, we collected 68 HFI patients, including the three patients mentioned above, by searching ClinVar, HGMD and published papers (Additional file 3: Table S3). These patients were from different populations. We analyzed the relationship between phenotype and variant site, mutation type, and zygosity. The onset of most symptoms was in infancy after weaning (94%, 15/16), and symptoms could be prevented by strict dietary restriction (100%, 21/21). The gastroenteric and liver phenotypes were the dominant phenotypes in the HFI patients. We found that 34 patients had an aversion to sweets and fruit (87%, 34/39), 31 had vomiting (78%, 31/40), 19 had nausea (54%, 19/35), and 22 had hepatomegaly (59%, 22/37). For each variant-phenotype analysis, we found that patients carrying A150P (homozygote) were more likely to present nausea than patients carrying a combination of other variant sites (*P* value < 0.05, Additional file 4: Table S4). No other variant-phenotype relationship was detected. The mutation types of the 68 HFI patients were classified into missense, frameshift, nonsense, splicing and in-frame indel types in our study (Additional file 4: Table S5). For mutation type-phenotype analyses, we found that patients carrying two missense variant sites were more likely to present aversion to sweets and fruit (*P* value < 0.05). For zygosity-phenotype analyses, patients carrying homozygous variant sites were more likely to present nausea (*P* value < 0.05) by the chi-square test (Additional file 4: Table S6). The other mutation type-phenotype and zygosity-phenotype relationships were not detected.

## Discussion

In this study, we estimated the prevalence of HFI in the Chinese population based on public and internal databases. We found that the HFI prevalence in the Chinese population is extremely low. However, poor prognosis of HFI can be easily avoided by special diet therapy, and patients benefit from early diagnosis. Therefore, we recommend that HFI testing be added to an expanded screening panel for neonatal and included into a diagnostic genetic testing panel for babies suspected of having HFI. The suspected phenotype includes frequent emesis, hypoglycemia and liver dysfunction after intake of baby formula (containing sucrose) [1].

We summarized the phenotype spectrum of 68 HFI patients and found that the digestive phenotype was the dominant feature. Therefore, for children, we suggested four typical symptoms in the workflow of HFI diagnoses: (1) vomiting and nausea after eating food or medicine containing fructose; (2) aversion to sweets and fruit; (3) abnormal liver function tests; and (4) symptoms that can be prevented by withdrawal of all sources of fructose. As fructose malabsorption (FM) and fructose-1,6-bisphosphatase deficiency (FBPase deficiency) have a similar phenotype to that of HFI, the onset of gastrointestinal symptoms in infancy after weaning can be prevented by strict dietary restriction. The differential diagnoses could be very difficult according to clinical phenotype. FBPase deficiency is an inherited AR disease caused by *FBP1* gene mutation [2]. Whole-exome sequencing (WES) can help identify which kind of fructose disease and exclude other metabolic diseases [18]. However, WES is not inexpensive enough to be used as a first-line diagnostic method. In addition, the symptoms of these three diseases can be easily controlled by fructose avoidance. Therefore, we tried to establish a cost-effective variant panel for HFI diagnosis. The rapid high-frequency site screening panel contains ten high-frequency sites, including A338V, N120Kfs*32, A338G, W296*, E225Rfs*5, R304W, A150P, c.325-1G > C, L289Ffs*10 and Q111*. These variants were identified as the top 10 variants with high AF in the CCGT pediatric cohort and covered approximately 90% of Chinese pediatric patients. By applying this panel test, time and expenditure could be reduced to a large degree. Nevertheless, the specificity and sensitivity need to be verified in large cohorts of healthy children.

HFI is a genetic metabolic disease, and digestive system symptoms are the dominant phenotype recognized by most researchers [19]. We diagnosed three patients, with gastrointestinal phenotypes. The genotype–phenotype association analyses were based on 68 reported HFI patients from a literature review and the CCGT database. By integrating the information from these 68 patients, we found that gastroenteric and liver phenotypes were the dominant phenotypes. A150P was identified as related to nausea. The function of A150P is unclear. The *ALDOB* sequence is generally highly conserved, but A150 is in a nonconserved area. Nevertheless, the detected relationship of A150P with nausea is a common relationship [8]; however, functional studies are needed to confirm this relationship. A150P is a hotspot in Caucasians but was not found in Chinese patients in this study. Two variants (A338V, A338G) had significantly higher AF in the Chinese population. We found two missense variant site patterns related to aversion to sweets and fruit in 68 HFI patients. We also found that homozygous variant sites were related to nausea. Two missense patterns (44/68, 65%) and homozygous patterns (35/68, 51%) were the main mutation types in the 68 HFI patients. Therefore, we thought these relationships may indicate the characteristics of *ALDOB* variants.

Fructose metabolism in the liver, kidney and intestine requires the synergistic action of two enzymes. In physiological states, fructokinase phosphorylates fructose into F-1-P, and aldolase B decomposes F-1-P into dihydroxyacetone phosphate and glyceraldehyde. Mutation of *ALDOB* could reduce the aldolase B activity and the accumulation of F-1-P, leading to deficiency in phosphate and adenosine triphosphate (ATP) and an increase in uric acid [19, 20]. The renal manifestation of HFI has been reported [6, 21–23]. This may indicate that the kidney could be damaged in HFI patients. One patient with proximal tubular acidosis has also been reported [24]. However, the characteristics of renal phenotypes of HFI have not been summarized.

CCGT is not a cohort of healthy individuals, and the genotype frequency associated with this cohort does not correspond with Hardy–Weinberg equilibrium. Therefore, we applied Bayesian analysis to combine the information on variants and overcome this shortcoming. This approach has a great advantage in genetic disease analyses across studies and calculates the prevalence of rare diseases in populations [25, 26]. We also performed analyses based on the HuaBiao and gnomAD-East Asian databases to mimic healthy and naturally gathered populations. The estimated prevalence was similar and indicated that CCGT could be used to present the genotype and phenotype characteristics of HFI. With more detailed information, we hope to update the HFI diagnosis strategy in the future.

## Conclusions

The prevalence of HFI in the Chinese population is extremely low, and there is no need to add HFI testing to the current newborn screening system if medical costs are considered. A genetic test strategy was suggested for early diagnosis of HFI, especially for patients with typical symptoms. The curated variant panel can be used to assist in making quick and inexpensive HFI diagnoses in China.

## Materials and methods

### Data acquisition for Chinese population data

This study was approved by the ethics committees of Children's Hospital of Fudan University (2021–464). The local CCGT cohort was the same as in our previous study, and the detailed processing steps can be found in that study [25]. Briefly, counseling was carried out, and informed consent was obtained from the parents of patients. Each individual received WES or clinical exome sequencing (CES), both covering the exon region and exon–intron splicing junction region (deep intron to 15 bp) of *ALDOB* genes. Both tests were sequenced on the Illumina HiSeq X10 with 150 bp paired-end sequencing. The genetic diagnosis of HFI was performed according to ACMG guidelines by experienced clinicians and genetic counselors [27, 28]. One parent was diagnosed with special requirements. For the HFI prevalence estimation, children and parents with a genetic diagnosis of HFI, together with their family members, were excluded.

### Literature search of HFI-related studies

PubMed and Web of Science were searched using the terms “Hereditary fructose intolerance”, “Hereditary fructose intolerance and case report”, “*ALDOB* mutation”, and “*ALDOB* variant” between 1988 (first described pathogenic variant) and October 2021 [15]. We applied strict literature inclusion criteria to make a more accurate conclusion. Our literature inclusion criteria were as follows: (1) the literature about case reports and the nomenclature of mutation sites meeting the requirements of HGVS [29, 30]; (2) the sites of case reports evaluated as P/LP according to ACMG guidelines; and (3) the literature included in SCI (representing high-quality literature). The exclusion criteria were as follows: (1) lack of information on mutation sites or the nomenclature of mutation sites; (2) lack of clinical information; and (3) repeated cases. According to those criteria, a total of 711 articles were found, of which 17 were included in this study [1, 5, 13, 23, 31–43].

### Curation of P/LP variants in the ALDOB gene

We included reported pathogenic variants of the *ALDOB* gene from ClinVar (level P or LP), HGMD (level DM or DM?) and the HFI-related literature mentioned above. No new variants were reported in the CCGT database. These variants were curated by two clinical geneticists back-to-back, and after manual checking, 81 out of 86 variants were curated at the P/LP level (Additional file 1: Table S1).

### Collection of the other population data

The AF of the *ALDOB* gene in other populations was available from the gnomAD. African American, admixed American, Ashkenazi Jewish, Finnish in Finland, non-Finland European, South Asian and East Asian populations in gnomAD were included (Additional file 1: Table S1). Gene annotation was from GENCODE, with ID (ENSG00000136872, ENST00000374855). GENCODE involves the annotation of functional elements in the human genome.

### Estimation of HFI prevalence in the Chinese population

We estimated the HFI prevalence by three strategies as described in our previous studies [25]. Method 1 was based on the carrier frequency of the individuals in the two cohorts calculated by the Hardy–Weinberg principle [15]. Method 2 was based on permutation and combination [25]. In this strategy, the hypothesis is to calculate the probability of an affected child by randomly choosing a male individual who carries the P/LP variant in the *ALDOB* gene and a female individual who also carries the P/LP variant in the *ALDOB* gene. Method 3 was based on the Bayesian framework with the gnomAD allele count dataset, where the 95% confidence interval could be estimated [44]. The third strategy was also adopted to estimate the HFI prevalence in other populations with only allele counts (Additional file 3: Table S3).

### Data acquisition and processing for the study of genotype–phenotype relationships

We collected the genotype and clinical characteristics of HFI patients from the CCGT database and HFI-related literature mentioned above to study genotype–phenotype relationships. After manual checking, 68 HFI patients were included (Additional file 3: Table S3). Here, the 24 common clinical manifestations were inferred from the OMIM database. The variants were further grouped by their mutation type and zygosity. Fisher’s exact test and the chi-square test were applied to test whether one phenotype was overrepresented in one type of mutation compared with the others.

### Statistical analyses

All statistical analysis was performed by R version 3.6.1. Chi-square test (λ2.test) was used for AF comparison. Multiple-test was adjusted by “bonferroni” strategy.

## Supplementary Information

**Additional file 1: Table S1.** Manually curated ALDOB variants’ pathogenicity. **Table S2.** Allele frequency for top 24 pathogenic variants to different populations.

**Additional file 2: Table S7.** Different populations from HuaBiao and gnomAD of HFI prevalence calculated based on the Bayesian framework. **Figure S1. **Ratio of predicted incidence.

**Additional file 3: Table S3.** Summary of genotype-phenotype information of 68 patients with HFI.

**Additional file 4: Table S4.** Relationship between variant site and phenotype. **Table S5.** Relationship between mutation type and phenotype. **Table S6.** Relationship between zygosity and phenotype.

## Data Availability

The data that support the findings of this study are either included in the article (or in its supplementary files) or available from the corresponding author on reasonable request. The data are not publicly available due to privacy or ethical restrictions.

## Abbreviations

HFI: Hereditary fructose intolerance
*ALDOB*: Aldolase enzyme, B isoform gene
AR: Autosomal recessive
CCGT: Chinese Children's Rare Disease Genetic Testing Clinical Collaboration System
AF: Allele frequency
HGMD: Human gene mutation database
CES: Clinical exome sequencing
P/LP: Pathogenic/likely pathogenic
VUS: Variant of unknown significance
ATP: Adenosine triphosphate
TAT: Turnaround time
WES: Whole-exome sequencing
