# Supplementary material for: Estimation of hereditary fructose intolerance prevalence in the Chinese population
Source: Orphanet J Rare Dis. 2022 Aug 26;17:326. doi: 10.1186/s13023-022-02487-3 (PMC9419342; doi:10.1186/s13023-022-02487-3)
Supplement: Supplementary file 2 — Additional file 2: Table S7. Different populations from HuaBiao and gnomAD of HFI prevalence calculated based on the Bayesian framework. Figure S1. Ratio of predicted incidence. [file 13023_2022_2487_MOESM2_ESM.docx]

**Supplementary Figures**

**
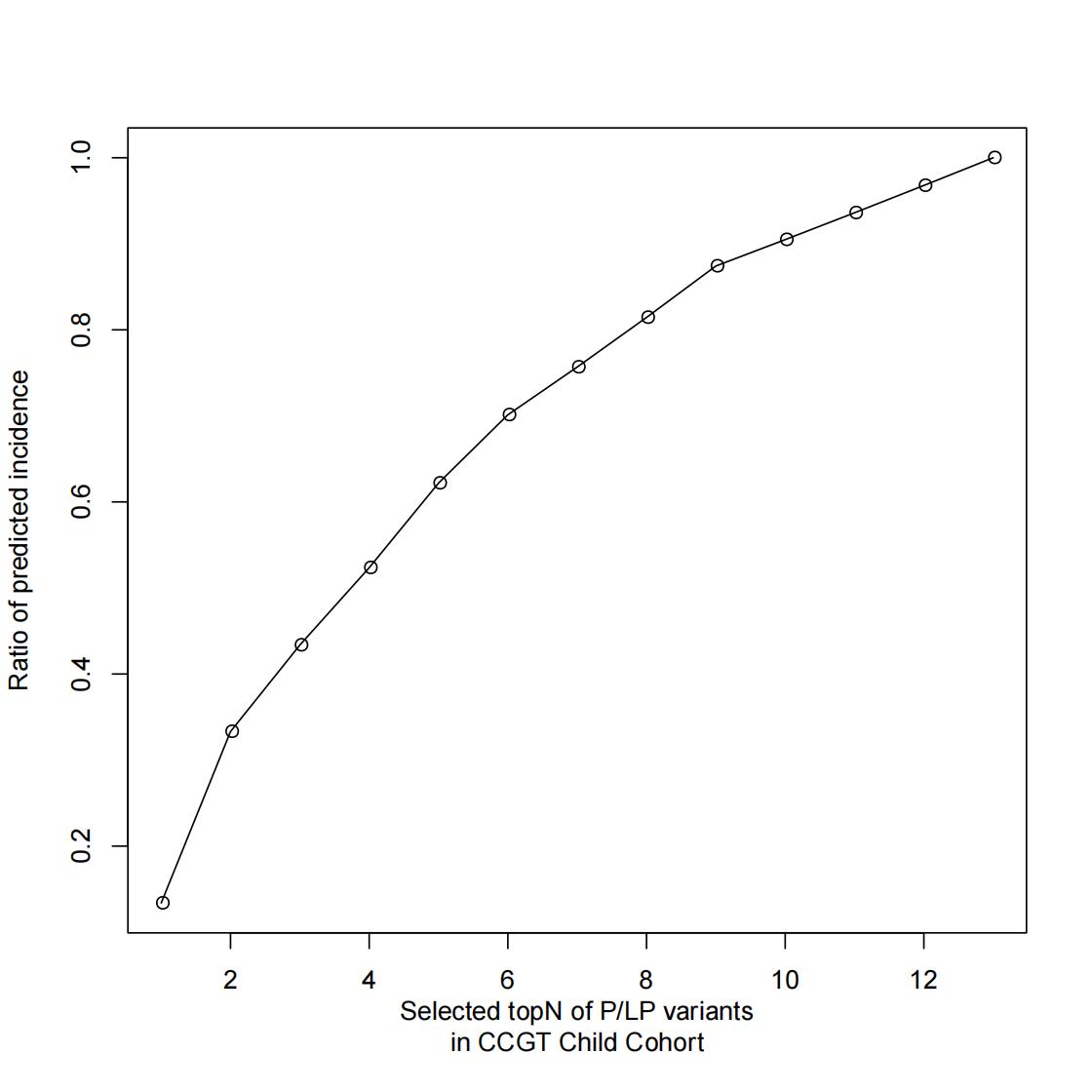
**

**Additional file 4: Figure S1. Ratio of predicted incidence.**

Estimated incidence based on 13 P/LP variants from CCGT Child Cohort. Rapid detection of the Top4 high-frequency sites (A338V, N120Kfs*32, A338G and W296*), which maybe cover about 50% of Chinese pediatric patients. Rapid detection of the Top10 variants (A338V, N120Kfs*32, A338G, W296*, E225Rfs*5, R304W, A150P, c.325-1G>C, L289Ffs*10 and Q111*) which maybe cover about 90% of Chinese pediatric patients.

**Supplementary Tables**

**Additional file 1: Table S1**. Manually curated *ALDOB* variants’ pathogenicity.

**Additional file 1: Table S2**. Allele frequency for top 24 pathogenic variants to different populations.

**Additional file 2: Table S3**. Summary of genotype-phenotype information of 68 patients with HFI.

**Additional file 3: Table S4**. Relationship between variant site and phenotype.

**Additional file 3: Table S5**. Relationship between mutation type and phenotype.

**Additional file 3: Table S6**. Relationship between zygosity and phenotype.

**Additional file 4: Table S7**. Different populations from HuaBiao and gnomAD database of HFI prevalence calculated based on the Bayesian framework.

| Population name | Estimation of HFI prevalence |
| --- | --- |
| HuaBiao | 1/410068(1/1395112-1/178484) |
| gnomAD-NFE | 1/23147(1/28182~1/19278) |
| gnomAD-FlN | 1/55539(1/107444~1/32816) |
| gnomAD-AMR | 1/132801(1/274861~1/75230) |
| gnomAD-ASJ | 1/263150(1/2275631~1/81267) |
| gnomAD-AFR | 1/412335(1/702621~1/264931) |
| gnomAD-SAS | 1/465278(1/7821624~1/121173) |
| gnomAD-EAS | 1/818758(1/25633515-1/188368) |
| gnomAD-TOTAL | 1/56399(1/66467~1/48326) |

Note: AFR: African American; ASJ: Ashkenazi Jewish; NFE: non-Finland European population; FIN: Finnish in Finland; AMR: admixed American population; SAS: South Asian; EAS: East Asian population.
